# Identification of a pore-forming protein from sea anemone *Anthopleura dowii* Verrill (1869) venom by mass spectrometry

**DOI:** 10.1590/1678-9199-JVATITD-1474-18

**Published:** 2019-02-11

**Authors:** Santos Ramírez-Carreto, Erick I. Pérez-García, Sandra I. Salazar-García, Johanna Bernáldez-Sarabia, Alexei Licea-Navarro, Enrique Rudiño-Piñera, Leonor Pérez-Martínez, Gustavo Pedraza-Alva, Claudia Rodríguez-Almazán

**Affiliations:** 1 Universidad Nacional Autónoma de México, Instituto de Biotecnología, Departamento de Medicina Molecular y Bioprocesos, Av. Universidad 2001, Cuernavaca, Morelos, México.; 2 Centro de Investigación Científica y de Educación Superior de Ensenada, Departamento de Innovación Biomédica, Baja California, México.

**Keywords:** Anthopleura, pore-forming protein, Venom, lung carcinoma, sea anemone

## Abstract

**Background:**

Pore-forming proteins (PFP) are a class of toxins abundant in the venom of sea anemones. Owing to their ability to recognize and permeabilize cell membranes, pore-forming proteins have medical potential in cancer therapy or as biosensors. In the present study, we showed the partial purification and sequencing of a pore-forming protein from *Anthopleura dowii* Verrill (1869). 17.

**Methods:**

Cytolytic activity of *A. dowii* Verrill (1869) venom was determined via hemolysis assay in the erythrocytes of four mammals (sheep, goat, human and rabbit). The cytotoxic activity was analyzed in the human adherent lung carcinoma epithelial cells (A549) by the cytosolic lactate dehydrogenase (LDH) assay, and trypan blue staining. The venom was fractionated via ammonium sulfate precipitation gradient, dialysis, and ion exchange chromatography. The presence of a pore-forming protein in purified fractions was evaluated through hemolytic and cytotoxic assays, and the activity fraction was analyzed using the percent of osmotic protections after polyethylene glycol (PEG) treatment and mass spectrometry. 18.

**Results:**

The amount of protein at which the venom produced 50% hemolysis (HU_50_) was determined in hemolysis assays using erythrocytes from sheep (HU_50_ = 10.7 ± 0.2 μg), goat (HU_50_ = 13.2 ± 0.3 μg), rabbit (HU_50_ = 34.7 ± 0.5 μg), and human (HU_50_ = 25.6 ± 0.6 μg). The venom presented a cytotoxic effect in A549 cells and the protein amount present in the venom responsible for producing 50% death (IC_50_) was determined using a trypan blue cytotoxicity assay (1.84 ± 0.40 μg/mL). The loss of membrane integrity in the A549 cells caused by the venom was detected by the release of LDH in proportion to the amount of protein. The venom was fractionated; and the fraction with hemolytic and cytotoxic activities was analyzed by mass spectrometry. A pore-forming protein was identified. The cytotoxicity in the A549 cells produced by the fraction containing the pore-forming protein was osmotically protected by PEG-3350 Da molecular mass, which corroborated that the loss of integrity in the plasma membrane was produced via pore formation. 19. Conclusion: *A. dowii* Verrill (1869) venom contains a pore-forming protein suitable for designing new drugs for cancer therapy.

## Background

Research on compounds derived from marine organisms has increased over the last four decades, and has focused on elucidating the mechanism of action of these compounds mainly to be applied in biomedicine [1]. The phylum Cnidaria is an ancient group with four main classes including corals, jellyfish, hydrozoans, and sea anemone [2,3]. The principal characteristic of this group is the production of nematocysts that are distributed in different tissues [4]. Nematocysts contain toxic compounds that are used against predators to protect their territory and to capture their prey [5].

The venom of cnidarians is composed of polypeptides such as cytolysins, protease inhibitors, proteases, neurotoxins, and phospholipases, and non-protein molecules such as serotonin and histamine [6,7]. The components of the venom have been determined by different strategies, e.g., electrical stimulation of the organism [8], isolation of nematocysts from the tentacles [9,10], and homogenization of the entire organism (extract) [11]. As a first step, to explore any potential applicability, the presence of toxins in the venom is identified by different bioassays [12,13] and the molecules with a potential specific function are purified [10,14]. Additionally, toxins immersed in the nematocyte matrix have been proven to target the plasma membrane [15,16]. Several sea anemone toxins have been studied for the development of drugs to serve as therapeutic agents with antitumor activity, as well as vasodilators, and ion channel modulators [17-22].

Among the different proteins identified from sea anemones are molecules capable of producing cytolysis. Cytolysins are classified into four groups based on their molecular weight and function as follows: 5 - 8 kDa antihistamine peptides, ~20 kDa pore-forming proteins or actinoporins, 30 - 40 kDa cytolysins with or without phospholipase A_2_ activity, and 80 kDa cytolysin isolated from *Metridium senile* with activity inhibited by cholesterol [23]. Actinoporins are the most studied cytolysin in sea anemones to date. These toxins form monomers in solution that binds to the membrane of a target cell, leading to pore formation [24-26]. Recently, the presence of a pre-pore was demonstrated as an intermediary in the action mechanism of actinoporins [27,28]. The pores produced by actinoporins alter the integrity of the membrane, producing an ionic imbalance that can lead to cell death [29,30]. The binding of actinoporins to the plasma membrane is determined by selective binding to sphingomyelin. This property is relevant to their use in cancer therapy [28,29] because it has been demonstrated that the lipids of the membranes of tumor cells present a significantly altered composition, particularly with a higher concentration of sphingomyelin [31,32]. The N-terminal region of actinoporins has an important role in the specificity of these proteins and can be internalized in the plasmatic membrane [33,34]. Several research groups have designed immunotoxins from the N-terminal of actinoporins [18,35], these conjugates can alter the cell membrane by producing cytotoxicity in tumor cells [37].

Lung cancer is one of the main causes of mortality worldwide [37,38]. Therefore, it is important to search for new compounds that have antitumor potential. In the present study, we analyzed the cytolytic and cytotoxic activities of venom from the sea anemone *A. dowii* Verrill (1869). We determined the amount of protein at which 50% of the erythrocytes were lysed (HU_50_) and at which 50% of A549 cells died. The hemolytic activity was assayed in erythrocytes from four mammals (sheep, goat, rabbit and human). The morphological changes of the A549 cells produced by the venom were observed by light microscopy. We suggest that the cytolytic effect was due to a pore-forming protein in the venom after analyzing the osmotic protectant effect of polyethylene glycol (PEG) and mass spectrometry. The cytotoxic activity was assayed in the A549 cell line (adenocarcinomic human alveolar basal epithelial cells), and was determined via trypan-blue-dye uptake and the lactate-dehydrogenase (LDH) release. 

## Methods

### Specimen collection

Four specimens of *A. dowii* Verrill (1869) were collected from the intertidal zone in Ensenada, Baja California, México. This species of sea anemone has been identified previously [39]. The organisms were transported to the laboratory where they were frozen and lyophilized, and then the samples were stored at -20°C until subsequent use. 

### Venom extraction

Forty milligrams of the lyophilized organisms was rehydrated with 125 mL of 50 mM sodium phosphate, pH 7.4, with protease inhibitor (Complement Protease Inhibitor Cocktail, Roche), after dissolving a protease inhibitor tablet in 1.5 mL of Milli Q water. The sample was mixed for 12 h at 4°C, using a magnetic stirrer to increase nematocyst discharge and release of toxins. The extract was subjected to three cycles of freezing and thawing at - 20°C [40]. The resulting solution was centrifuged at 25,400 x g for 40 min to remove cell debris. The supernatant was maintained at -20°C until use. Before use, the venom was centrifuged at 8,000 x g and filtered through membranes with a 0.22 μm pore size. The total protein concentration of the venom was determined via the Bradford assay [41]. The electrophoretic profile was examined by Tricine-SDS-PAGE on 15% polyacrylamide gels [42]. The gels were dyed with Coomasie blue.

### Hemolytic activity assay

The hemolytic activity of the venom was detected by the hemoglobin of erythrocytes released in response to different venom protein concentrations. Two milliliters of peripheral blood of each organism (human, rabbit, sheep, and goat) was collected and mixed with anticoagulant solution. The human, sheep, rabbit, and goat erythrocytes were washed three times with Alsever solution (0.1 M dextrose, 0.07 M NaCl, 0.002 M citric acid, and 0.027 M Sodium citrate, pH 7.4). The assay was performed in 96-well microtiter plates, with each well containing 20 μL of erythrocyte solution (1 - 2 x 10^6^ cells/mL), a different protein concentration, and for the anticoagulant solution an appropriate volume was added to adjust each well to 200 μL. The samples were incubated at 37 °C for 60 min and subsequently centrifuged at 804 x g for 5 min at 4°C. The amount of hemoglobin was spectrophotometrically monitored at λ = 415 nm and the hemolysis percentage was calculated using the following equation:

$$\%Hemolysis=(A_{V}-A_{buffer})/\left(A_{water}-A_{buffer}\right)x\;100$$

where A_V_, A_buffer_, and A_water_ are the absorbances (A) of the supernatants of venom (V), buffer (0% hemolysis), and water (100% hemolysis), respectively [43]. All hemolysis assays were performed in triplicate. The HU_50_ thus calculated represents the protein concentration from venom that produced 50% hemolysis. 

### Human cell culture

The human adherent lung carcinoma epithelial cells A549 were obtained from the American Type Culture Collection, USA. The cells were maintained in Gibco^TM^ Dulbecco´ s Modified Eagle Medium: Nutrient Mixture F-12 (DMEM-F12), supplemented with 5% fetal bovine serum, 2 mM L-glutamine, 50 U/mL penicillin, and 50 mg/mL streptomycin. In all experiments that included the A549 cell line, the cells were seeded in 24-well plates at 4 x 10^4^ cells/mL and were maintained at 37 (C in 5% CO_2_ for 48 h. After this, an average of 2 x 10^5^ cells per well were exposed to the venom and then incubated for 6 h at 37 °C and 5% CO_2_. 

### Cell viability assay

Cell viability assay was determined by trypan blue staining [44]. The cells were treated with trypsin, washed with 1X phosphate buffer saline (PBS), and resuspended in 1 mL of 1X PBS. Finally, cells were stained with trypan blue dye, with unstained cells counted as viable cells. The amount of protein present in the venom responsible for producing 50% death (IC_50_) in A549 cells was calculated through an adjusted mathematical model based on Boltzmann’s sigmoidal equation. The morphological changes in the cells were observed by light microscopy (110x magnification) after 6 h of exposure to the venom. 

### Cytotoxicity assay

The cytotoxic activity of venom in the A549 cells was monitored by cytosolic LDH release [45]. Controls were considered the supernatants of cultured cells without venom (0%) and cells were treated with 1% Triton X-100 (100%). LDH activity was identified in the supernatants using the CytoTox 96® Non-Radioactive Cytotoxicity Assay Kit (Promega Corporation, Madison, USA). The reaction was incubated at 25 °C in total darkness for 30 min. LDH activity was measured by absorbance at 490 nm using an iMarK Microplate Reader microplate reader (Bio-Rad). The percentage of cytosolic LDH released was calculated via the following equation:

$$\%LDH=({LDH}_{obs}-{LDH}_{0\%})/\left({LDH}_{100\%}-{LDH}_{0\%}\right)x100$$

### *Anthopleura dowii* venom fractionation

A total of 720 mg of protein contained in 200 mL of the crude extract was precipitated with an ammonium sulfate gradient (40%, 50%, 60%, and 80% of saturation) in 50 mM phosphate buffer at pH 7.4 and 4 °C [46]. Each gradient was kept cold overnight and centrifuged at 25,400 x g for 140 min at 4 °C. Each precipitate was resuspended in 50 mM phosphate buffer, pH 7.4, and dialyzed against the same buffer. To remove the non-soluble protein, the dialyzed samples were centrifuged at 25,400 x g for 10 min at 4°C. The samples were analyzed in 15% SDS-PAGE polyacrylamide gel [47] and their hemolytic and cytotoxic activities were measured. The fraction with the highest hemolytic and cytotoxic activities was applied to 10mL DEAE Sepharose Fast Flow anion exchange chromatography (GE) column and equilibrated with 50 mM phosphate buffer at pH 8.0. Elution was carried out with a step gradient of NaCl (0, 20, 40, 60, 80, 100, 200, 300 and 500 mM), at a flow of 1.0 mL/min. The fractions were subjected to an electrophoretic assay in 15% polyacrylamide SDS-PAGE gel, and their hemolytic and cytotoxic activities were measured. The gel was dyed with Coomasie blue.

### Osmotic protection assay

The osmotic protection assay with PEG was evaluated in A549 cells [48-50]. PEG (30 mM as final concentration) of different molecular sizes (200 Da, 400 Da, 1,000 Da, and 3,350 Da) and prepared in 1X PBS solution. The cells (2 x 10^5^ cells/mL) with PEG were incubated at 37 °C and 5% CO_2_ for 15 min, and then treated with 50μg of protein. After 6 h of incubation, the supernatant was collected and the LDH released was calculated as mentioned above. 

### Sample preparation for mass spectrometry

To remove the PBS, the sample was passed through a Sep-Pak C18 cartridge (Waters Corp., USA) and lyophilized. Subsequently, it was resuspended in 100 mM ammonium bicarbonate (pH 8 - 8.5) and quantified in triplicate in a NanoDrop 2000 (Thermo Scientific) at 280 nm. The cysteine reduction was performed at 10 mM DTT for 60 min at 37°C, and alkylation was performed with 20 mM iodocatamide for 30 min in darkness at room temperature. For digestion, trypsin (Promega) was used in a 1:40 w/w ratio for 4 h at 37 °C; Protease MAX (Promega) was added to the solution to improve solubility and promote digestion. Finally, the sample was desalted using a Sep-Pak C18, concentrated by lyophilization, and resuspended in water with 0.1% formic acid.

### Mass spectrometry

Analysis by liquid chromatography was performed in a HALO C18 column of 0.3 x 150 mm in the high-performance liquid chromatographer Eksigent NanoLC 400 (AB SCIEX Dublin, CA) coupled to the mass spectrometer TripleTOF 5600+ (MA, AB SCIEX Framingham, MA, USA) equipped with a DuoSpray font. The Analyst TF 1.7 program was employed to operate the equipment and acquire the data, which were collected in positive mode by electrospray ionization by applying the IDA method (information-dependent acquisition method). In the Mass spectrometry (MS), a mass range of 250 - 1500 was used and for the tandem MS (MS/MS), a range of 50 - 1500 m/z was utilized. The scan lasted 120 min with a cycle time of 1.3 s and a total of 5,536 cycles. In each cycle, the maximum number of monitored ions was 20 with a tolerance of 0.1 Da. In total, two injections were made, one of 2 μg and another of 6 μg. The mobile phases consisted of water with 0.1% formic acid (A) and acetonitrile with 0.1% formic acid (B). The separation was performed by a linear gradient of 5% - 30% B for 110 min and 30 - 100% B for 10 min with a flow of 5 μL/min.

### Protein identification

The data obtained directly from the MS were processed by the software ProteinPilot 4.5 (AB Sciex) using the Paragon algorithm to search for matches within the UniProt Anthopleura (230 entries) and Actiniidae (708 entries) databases. Trypsin was considered a digestion enzyme, idoacetamide a cysteine ​​alkylating agent, and identification was established at > 95% probability.

### Statistical analysis

The data are expressed as the mean ± SD of three different experiments with triplicate values. An analysis of variance was utilized for the statistical analysis, and a Turkey’s multiple comparison test was performed via the software OriginPro (version 9). Differences were considered statistically significant at p values less than 0.05.

## Results

### Hemolytic and cytotoxic activities of *A. dowii* venom

**Figure 1:**
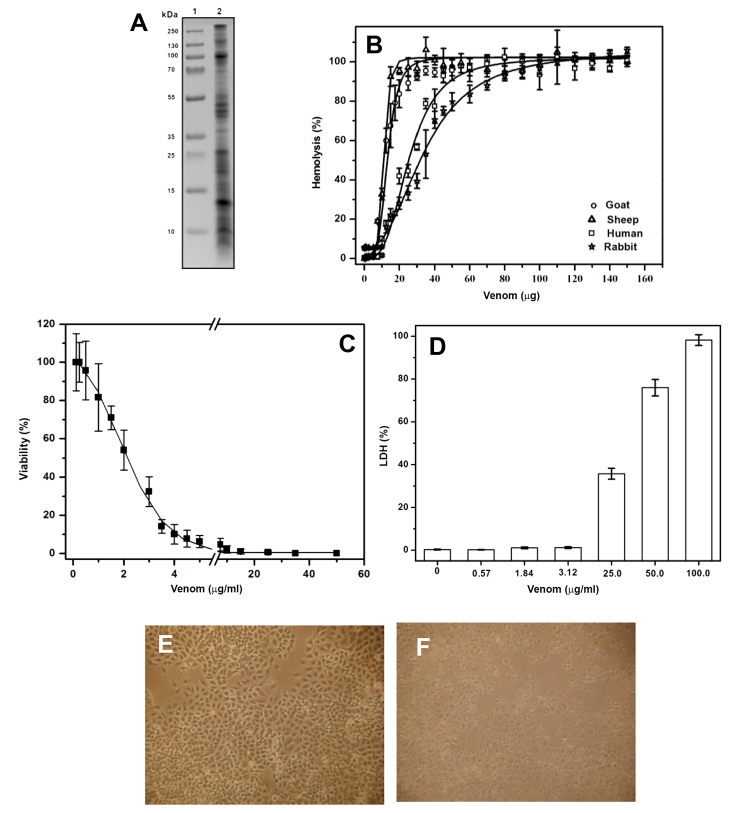
Characterization of *A, dowii* Verrill (1869) venom. **A.** SDS-PAGE analysis of venom, lane 1: protein molecular weight marker, lane 2: venom. **B.** Hemolysis produced by the venom in erythrocytes from different mammals. **C**. Viability assay of A549 cells exposed to different concentrations of sea anemone venom. **D**. Cytotoxicity was analyzed by measuring LDH released from A549 cell line. Morphological changes of A549 cells without (**E**) and with (**F**) 50 μg/mL of total protein from venom were observed via transmitted light microscopy.

The presence of polypeptide molecules that exhibit diverse biological activities such as hemolytic and cytotoxic capacities present in sea anemone venom has been reported previously [19,51]. The extraction method employed to prepare the *A. dowii* venom allowed us to obtain a mixture of different molecules, including polypeptides with a molecular weight between 10 kDa and 250 kDa. We detected proteins with molecular weights similar to those reported for actinoporins (~20kDa) [15] (Figure 1A). To identify the cytolytic activity in the venom of *A. dowii*, a hemolytic assay was performed on human, rabbit, sheep, and goat erythrocytes. Our results showed that the venom has components capable of producing lysis in erythrocytes of these four mammalian species. In all cases, hemolysis tended to rise as the concentration of total protein present in the venom increased until 100% lysis. Hemolytic activity showed a significant difference between goat and sheep erythrocytes compared to human and rabbit erythrocytes. Thus, sheep erythrocyte membranes were significantly more susceptible to the hemolytic activity of the venom with a HU_50_ equal to 10.7 ± 2 μg, whereas 50% of the goat, human and rabbit erythrocytes were lysed with 13.2 ± 3 μg, 25.6 ± 0.5 μg and 34.7 ± 0.6 μg, respectively (Figure 1B). According to previous studies, these differences in hemolytic activity may be due to the lipid composition of the membranes from different red blood cells [24,27].

It has previously been demonstrated that the cytoplasmic membrane of tumor cells has a significantly altered composition compared to normal cells, with higher concentrations of sphingomyelin, phosphatidylinositol, and cholesterol [31,52]. Some cytolysins from sea anemones, such as actinoporins, have a high affinity for lipids including sphingomyelin or cholesterol, which is essential for its action mechanism [24,53]. To investigate the effect of venom on human alveolar adenocarcinoma (A549 cell line), cytotoxicity assays were performed on A549 cells with an increasing concentration of venom, and the number of living cells was determined by the trypan blue exclusion method. The venom decreased viability in a dose-dependent manner, in a protein concentration range of 0.125 - 50 μg/mL. The protein concentration in the venom responsible for producing 50% death (IC_50_) was equal to 1.84 ± 0.4 μg (Figure 1C). In the presence of venom, contact between the cells decreased, and their polygonal form with membrane protrusions changed to a round shape. Figure 1E and 1F show representative images of the experiments where the morphological changes of the cells are clearly observed in the presence of 50 μg of total protein in the venom. 

To determine whether the venom promoted cell death by causing damage to the plasma membrane, the release of LDH was measured. LDH is a stable enzyme that is removed from the cells in high quantities after plasma membrane damage has occurred, and is an indicator of necrosis [45]. Our results showed that this phenomenon is concentration-dependent. At 3.12 ± 0.40 μg/mL extracellular LDH was not detected. However, concentrations of 25, 50, and 100 μg/mL of total protein caused LDH release (Figure 1DD). These results suggest that at higher protein concentrations in the venom, the membrane integrity is compromised. These results indicate that *A. dowii* venom contains different components that induce cell death by two different mechanisms; i.e., at low concentration it could indicate apoptosis, whereas at high concentrations it may represent cell lysis. 

### *A. dowii* venom fractionation

**Figure 2:**
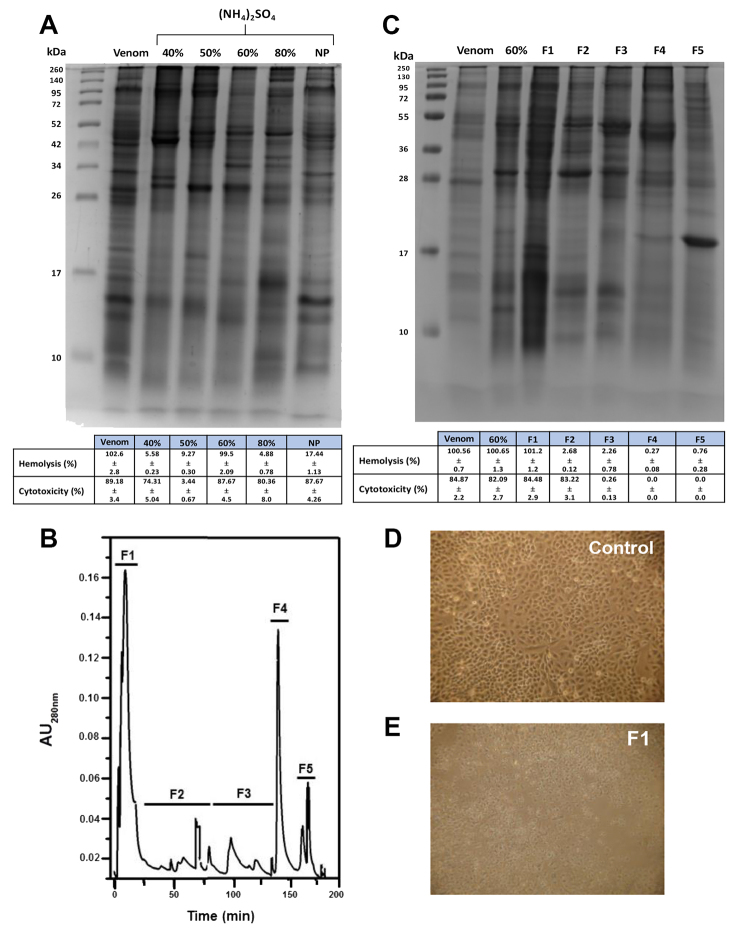
Venom fractionation. **A**. Gradual precipitation of venom with ammonium sulfate. SDS-PAGE, hemolytic activity in human red blood cells, and cytotoxicity in A549 cells determined by released LDH. **B**. Anion exchange chromatography of proteins precipitated with 60% ammonium sulfate. Five fractions were obtained by chromatography (F1: 0 mM NaCl, F2: 20 - 60 mM NaCl, F3: 80 - 100 mM NaCl, F4: 200 mM NaCl, F5: 300 mM NaCl). **C**: Denaturing electrophoresis of the fractions obtained in B, hemolytic activity in human erythrocytes and cytotoxicity in A549. **D** and **E**: A549 cells without and with F1, respectively.

Venom was precipitated with a gradient of ammonium sulfate. Figure 2A shows the electrophoretic pattern, and the hemolytic and cytotoxic activities of each fraction. The proteins precipitated with 60% ammonium sulfate were the only fraction that showed hemolytic activity. Apart from the proteins precipitated with 50% ammonium sulfate, the rest had cytotoxic activity. The morphology of A549 was also affected by these samples (see Additional File 1). The fraction with hemolytic activity was applied to an anion exchange column and eluted by a NaCl gradient (Figure 2B). Most proteins did not bind to the column. All fractions were analyzed in a 15% polyacrylamide gel SDS-PAGE and their activities were measured. The F1 fraction was the only one that presented hemolytic and cytotoxic activities (Figures 2C, 2B, and 2E). F1 and F2 fractions (Figure 2C) produced similar morphological changes in A549 cells and LDH in relation to those observed with whole venom (see Additional File 1). 

### Damage to plasma membrane in the A549 cell line by pore formation

**Figure 3:**
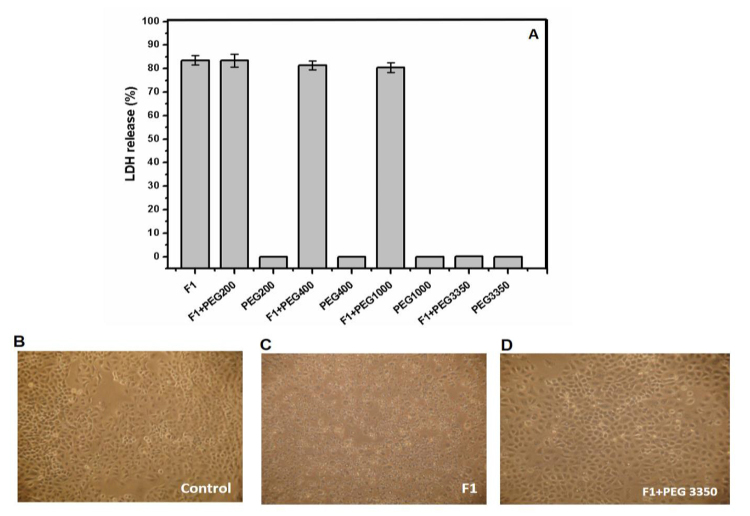
PEG as osmotic protectants in A549 cells in the presence of F1 fraction. **A**. Cells were previously incubated with different PEG before adding 50 μg of F1 fraction. The buffer used was 50mM phosphate, pH 7.4. **B, C and D**: Damage to the cells by 50 μg/mL of the F1 fraction was inhibited by the addition of PEG 3,350.

Sea anemones produce a class of proteins that can alter the cell membrane through pore-formation that provokes cellular lysis by osmotic imbalance [54]. This can be countered by the use of osmotic protectants such as PEG [55,56]. To determine whether proteins from the F1 fraction produced damage to A549 cells because of pore formation, the percent of osmotic protection was determined based on LDH released after PEG treatment in the presence or absence of 50 μg of total protein. As shown in Figure 3A, osmoprotectant PEG 200 Da, 400 Da, and 1,000 Da were significantly different compared with PEG 3,350 Da. The LDH activity was reduced by 100% only in the presence of PEG 3,350 Da. As shown in Figures 3B, 3C and 3D, the osmotic protection from PEG in the presence of the F1 fraction derived from A549 cells preserved their polygonal morphology. This result shows that the cytotoxic activity of the F1 fraction could be due to the presence of pore formation. To corroborate this, F1 was analyzed by mass spectrometry to identify the presence of a pore-forming protein. 

### Polypeptides identified in F1 fraction by MS / MS

**Figure 4:**
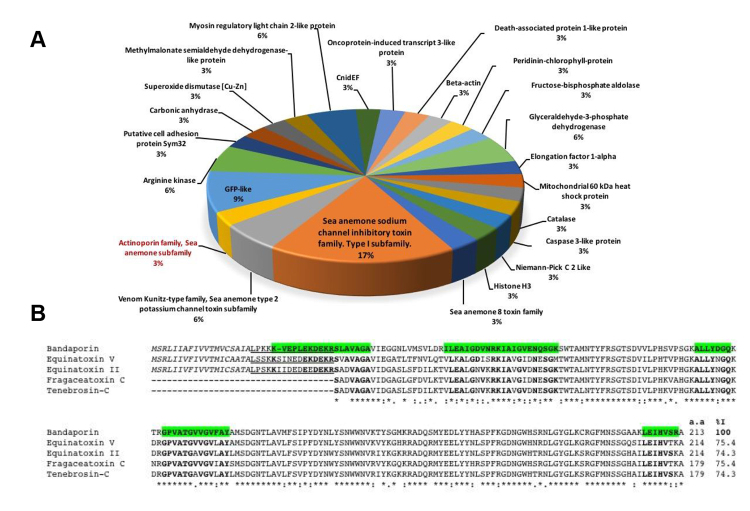
Distribution of the components identified by MS / MS in the F1 fraction and multiple alignment of actinoporins showing the region covered by the tryptic peptides. **A.** Relative proportions of the polypeptides identified using the *Anthopleura* and Actiniidae databases from UniprotKB. **B.** Multiple alignment of bandaporin (DELTA-actitoxin-Aas1a) from *Anthopleura asiatica*, Equinatoxin II (DELTA-actitoxin-Aeq1a) and V (DELTA-actitoxin-Aeq1b) from *Actinia equina*, Fragaceatoxin-C (DELTA-actitoxin-Afr1a) from *Actinia fragacea* and Tenebrosin-C (DELTA-actitoxin-Ate1a) from *Actinia tenebrosa*. The region covered by the triptych peptides in the bandaporin sequence is highlighted in green. In all sequences, the residues conserved with the region covering the tryptic peptides appear in boldface. The signal peptide is in italics and the propeptide region is underlined.

The F1 fraction was processed by MS to explore its polypeptide composition. Data were processed by the software ProteinPilot 4.5 (AB Sciex) using the cured (UniprotKB/Swiss-Prot) and uncured databases (UniprotKB / TrEMBL) of *Anthopleura* (https://www.uniprot.org/uniprot/?query=Anthopleura&sort=score) with 230 total entries and Actiniidae with 708 total UniProt entries during September 2018 (https://www.uniprot.org/uniprot/?query=Actiniidae+&sort=score). A total of 35 polypeptides with molecular weight between 4 and 80 kDa were identified in the protein sample from the tryptic peptides from F1 (see Additional Files 2 and 3). The identified proteins were grouped according to their function and their relative abundances were calculated (Figure 4A). The components mostly represented in the F1 fraction corresponded to Na+ toxins (17%). Other components related to the sea anemone venom included K+ toxins with inhibitory activity of Kunitz type proteases and toxins of family 8 (see Additional File 2). Only one actinoporin was identified in the F1 fraction, for which 32% coverage was obtained with respect to DELTA-actitoxin-Aas1a (Figure 4B). The remainder of the proteins identified in the F1 fraction were proteins not related to toxic function and those that have been reported in other species of sea anemones, such as enzymatic proteins (e.g., superoxide dismutase, carbonic anhydrase), enzymes related to the metabolism of carbohydrates, arginine kinases, structural proteins, and adhesion and stress proteins.

At least 13 tryptic peptides that align with regions of the reported protein such as bandaporin (DELTA-actitoxin-Aas1a) were identified in the F1 fraction (see Additional File 2) covering 32% of the amino acid sequence. Bandaporin has previously been isolated from *A. asiatica* whereas its hemolytic activity on sheep erythrocytes (EC_50_ of 8.8 ng/mL) and lethal toxicity (LD_100_ equal to 0.58 mg/kg) were estimated in crayfish [57]. Figure 4B shows the alignment of bandaporin with four other actinoporins. The region covered by the triptych peptides was identified (Figure 4B, green colored region) in the F1 fraction, which showed 100% identity with the bandaporin sequence with respect to the other four actinoporins present in the alignment. The identity percentage (% I) was calculated via the software LALIGN (https://embnet.vital-it.ch/software/LALIGN_form.html) considering only the mature regions of all proteins. Alignment was accomplished using the software ClustalX (2.0). 

## Discussion

Sea anemone venom is a complex mixture of molecules with different targets and diverse biological functions (e.g., neurotoxins, phospholipases, cytolysin, protease inhibitors, antimicrobial peptides, and lectins). The main interest in the investigation of these molecules is to isolate and characterize candidates potentially useful for the development of new drugs [1,7]. The present study showed the identification of a pore-forming protein in the *A. dowii* venom. Among proteins previously described in sea anemones to have pore-forming capacity, actinoporins stand out owing to their proven ability to form pores in the cell membrane, which are directly responsible for osmotic imbalance and cell death [15,58]. The membrane-binding activity of actinoporins depends on the lipid composition, while a high concentration of sphingomyelin has been proven to facilitate pore formation [24,59,60]. The lipid compositions differ significantly among goat, sheep, rabbit and human erythrocytes, especially in their sphingomyelin content [61,62]. The hemolytic activity results produced by the *A. dowii* venom showed that the erythrocytes with the highest amount of sphingomyelin were from the sheep, which were more sensitive to the cytolytic effect produced by proteins in the venom. It has recently been reported that the activity of four actinoporins (equinatoxin II, fragaceatoxin C and sticholysins I and II) have different activities that present variations in the composition of lipids and biophysics membrane [24].

One method of studying *in vitro* cytotoxicity is by measuring LDH leakage. This assay indicates the permeability increase of the membrane as a consequence of damage to its structure, resulting in the release of intracellular components and subsequent cell death [63]. The release of intracellular LDH demonstrated that the protein components of the venom and F1 fraction were capable of inducing A549 cells lysis. Further studies are required to determine the mechanism by which cell death occurred. In the sea anemone *Heteractis magnifica* venom was reported to produce necrosis in A549 cells, and this was determined by mitochondrial membrane potential [12]. The A549 cell viability was reduced by approximately 70% when using 3.5 μg of *A. dowii* venom as determined by the trypan blue exclusion assay. The fact that at this concentration no LDH activity was detected in the supernatants indicates that, in addition to cell membrane damage and cytolysis, the *A. dowii* venom was able to induce cell death by a distinct mechanism, but this must still be tested. The morphological changes observed in A549 cells are similar of those observed in Raji and L1210 cells exposed to Sticholysin II and to Nigrelysin respectively [64,65], these two actinoporins cause an increase in cell volume, loss of polygonal shape and subsequent lysis. However, a cell death process, such as pyroptosis or the phenomenon of cell lysis, can be an independent event, as has been reported in macrophages from mouse bone marrow, in which pyroptotic cell death was observed to be an independent event preceding cell lysis [66]. 

Owing to the presence of properties that can recognize and permeabilize plasma membrane, these pores have a medical potential and application in challenging cell proliferation such as in cancer therapy. Based on previous studies, it has been proposed that specific immunotoxins be constructed using a conjugate of monoclonal antibodies and cytolysin from sea anemones [36]. The main advantage of immunotoxins composed of anemones cytolysins is that they do not need to be applied directly in the intracellular space to destroy a particular neoplasm [18,36]. 

The integrity of the plasma membrane is crucial for proper cellular function and stability. The pores produced by actinoporins produce an ionic imbalance or uncontrolled flow and permeabilization to a variety of molecules that depend on the pore size [55,67]. Our results have shown that damage to the plasma membrane of A549 cells may be the result of pores formed by proteins. The osmotic lysis generated by the formation of pores in the plasma membrane can be avoided by the use of osmolytes such as PEG, which prevent cytoplasmic components such as LDH from being released [40]. Our results allowed us to observe a remarkable reduction of lysis in A549 cells by adding PEG of 3,350 Da, whereas the damage to the cell membrane appeared to occur through the action of a pore-forming protein present in the *A. dowii* venom and F1 fraction. These results are consistent with the fact that PEG can inhibit the hemolysis produced by pore-forming proteins [68]. 

Because of the complexity of the *A. dowii* venom shown in the data obtained during fractionation, we propose a more detailed analysis of the fractions obtained in the precipitation with ammonium sulfate (40%, 80%, and not precipitated), principally fractions with high cytotoxic activity in A549 cells, and very low hemolytic activity. This indicates the presence of more than one molecule with possible antitumor potential in *A. dowii* venom.

## Conclusions

*A. dowii* venom contains at least one polypeptide with the ability to lyse cells by the pore-formation mechanism in the plasma membrane of the A549 cell line. This molecule should be considered in the design of drugs for cancer therapy, with future investigations required to analyze their action mechanism.

### Abbreviations

HU_50_: the protein concentration in the venom that produced 50% hemolysis.

IC_50_: the amount of protein present in the venom responsible for producing 50% death in A549 cells.

## Supplementary material

The following online material is available for this article

Additional File 1: The morphological change in A549 cells is demonstrated by the protein precipitation with different concentrations of ammonium sulfate (A) and with the fractions obtained in the anion exchange chromatography (B).(pdf 113 kb)

Additional File 2: Peptides and proteins identified in the F1 fraction of the venom of *Anthopleura dowii* using LC - MS/MS. (pdf 138kb).

Additional File 3: ProteinPilot v 4.5 software program (AB Sciex) report of the tryptic peptides obtained from the F1 fraction (2 μg and 6 μg analyzed) and used for the identification of the protein components present in the sample using the Anthopleura and Actiinidae cured databases (UniprotKB/Swiss-Prot).
