# Supplementary material for: Identification of a pore-forming protein from sea anemone Anthopleura dowii Verrill (1869) venom by mass spectrometry
Source: J Venom Anim Toxins Incl Trop Dis. 2019 Feb 11;25:e147418. doi: 10.1590/1678-9199-JVATITD-1474-18 (PMC6483413; doi:10.1590/1678-9199-JVATITD-1474-18)
Supplement: Supplementary file 1 [file 1678-9199-jvatitd-25-e147418-s1.pdf]

# Supplementary Material to "Identification of a pore-forming protein from sea anemone *Anthopleura dowii* Verrill (1869) venom by mass spectrometry"

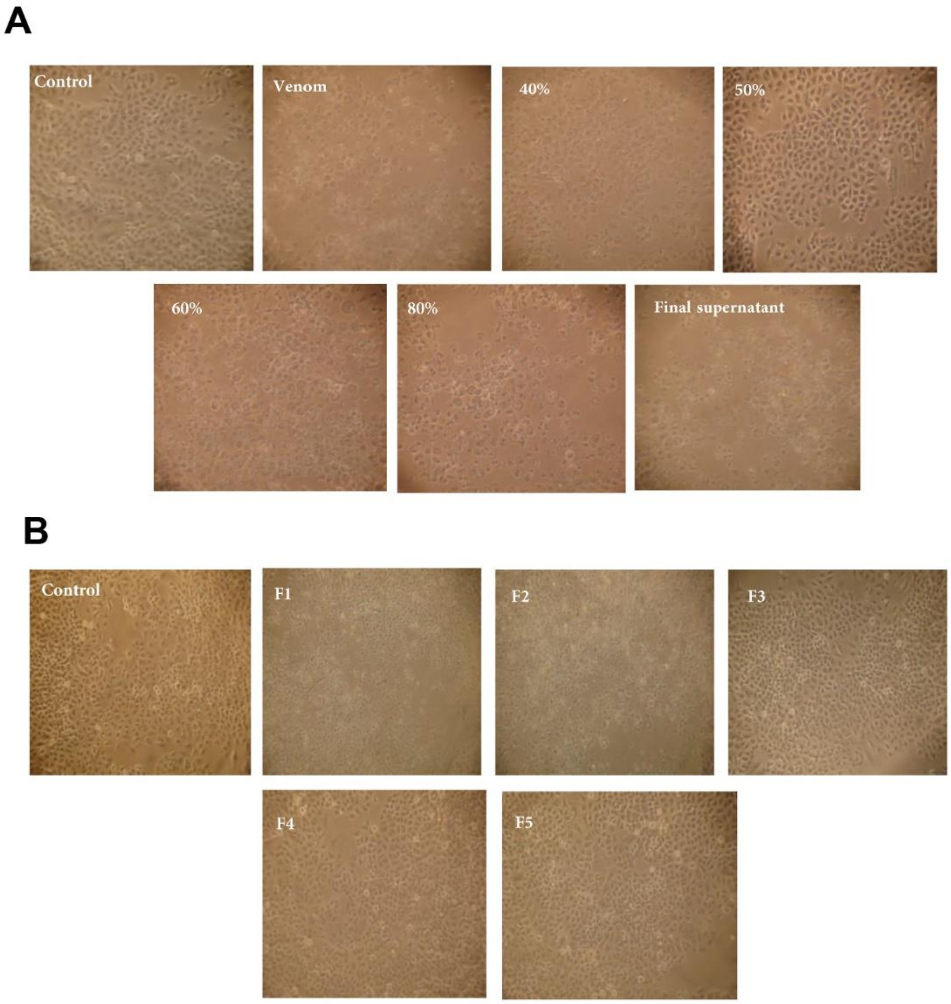


**Additional File 1:** The morphological change in A549 cells is demonstrated by the protein precipitation with different concentrations of ammonium sulfate (A) and with the fractions obtained in the anion exchange chromatography (B).
