# Supplementary material for: Identification of a pore-forming protein from sea anemone Anthopleura dowii Verrill (1869) venom by mass spectrometry
Source: J Venom Anim Toxins Incl Trop Dis. 2019 Feb 11;25:e147418. doi: 10.1590/1678-9199-JVATITD-1474-18 (PMC6483413; doi:10.1590/1678-9199-JVATITD-1474-18)
Supplement: Supplementary file 2 [file 1678-9199-jvatitd-25-e147418-s2.pdf]

# Supplementary Material to "Identification of a pore-forming protein from sea anemone *Anthopleura dowii* Verrill (1869) venom by mass spectrometry"

**Table 1.** Peptides and proteins identified in the F1 fraction of the venom of *Anthopleura dowii* Verrill (1869) using MS / MS.

| Name | UniProtKB[a]/ Entry name | Organism | Family/ Scafold[b] | Coverage (%) | Mass (Da)[c] | Sequences of the polypeptides identified from the tryptic peptides obtained by MS /MS.[d] |
|---|---|---|---|---|---|---|
| Arginine kinase | O15992/ KARG_ANTJA | *Anthopleura japonica* | ATP:guanido phosphotran sferase family | 37 | 80,024 | MADPETAAKFKSKNAFPDPLNDPKCNPKSLVKKYLTPKVFESLKNKKTKLGITL<br>WDCINSGVVNLDSGVGVYAGDEESYTLFGPLFDAIIEDYHSPYKLATGHNSDM<br>NPAHVKAPDLDPANRYIRSTRIRVARSLKGYGLAPGVTKAHRLEIEKKVVGVLT<br>SLTGDLAGKYYPLSGMDEKTRQQLVDDHFLFKKGDRFLEAAGINKEWPEGRGI<br>YHNNDKTFLVWLNEEDHLRIISMEKGSDIGSVFSRLCRAVNEIDKKLGFQHTKK<br>HGYLTSCPSNLGTGMRASVHVKIPHAKEHPDFENILTKYHIQARGIHGEHSEST<br>GEDAGVYDISNRRRLGLSEVQCVQDMYDGVKALMELEKEAIAKKRSVFPEVLK<br>NPEVKSLLRKYLTPELFDSLKDKKTAKGISLYDCINSGVENLDSSCGVYAGDEEC<br>YTLFAPLFDKIVEDYHSPYKLANKHTSDMNPEKVDAPNLDPEGTYIRSTRIRVAR<br>NVKGYALTPGLTRNERLDIERKVVGVLSSLTGDLAGQYYPLTGMDEATRQKLV<br>NDHFLFKKGDRFLEAAGVNKLWPEGRGIFHNNDKTFLVWINEEDQLRIISMEK<br>GSDIGSVFGRLCRAVNEIDKQLGFQHTDAHGYLSGCPTNLGTGMRASVHVKIP<br>KASAHPDFQKICDEFHIQARGIHGEHSVSTGEDAGVFDISNRRRLGLSEVQCV<br>QDMYNGVKKLLEIEKSTK |
| Arginine kinase (Fragment) | Q5QH95/ Q5QH95_ANTXA | *Anthopleura xanthogrammica* | ATP:guanido phosphotran sferase family | 24 | 6,612 | SNRRRLGLSEVQCVQDMYNGVKKLLEIGQAAIQEKRSKFPEGLKDSSVKSLLKK<br>YLTE |

| Name | UniProtKB[a]/ Entry name | Organism | Family/ Scafold[b] | Coverage (%) | Mass (Da)[c] | Sequences of the polypeptides identified from the tryptic peptides obtained by MS /MS.[d] |
|---|---|---|---|---|---|---|
| Delta-actitoxin-Axm1a | P01530/ NA1A_ANTXA | *Anthopleura xanthogrammica* | Sea anemone sodium channel inhibitory toxin family. Type I subfamily. | 100 | 5,138 | GVSCLCDSDGPSVRGNTLSGTLWLYPSGCPSGWHNCKAHGPTIGWCCKQ |
| Delta-actitoxin-Ael1b | P0C1F0/ NA11_ANTEL | *Anthopleura elegantissima* | Sea anemone sodium channel inhibitory toxin family. Type I subfamily. | 100 | 4,803 | GIACLCDSDGPSVRGNTLSGTYWLAGCPSGWHNCKSSGQLIGACCKQ |
| KappaPI-actitoxin-Ael3a | P86862/ VKT1_ANTEL | *Anthopleura elegantissima* | Venom Kunitz-type family. Sea anemone type 2 potassium channel toxin subfamily. | 73 | 7,475 | INSICLLPKKQGFCRARFPRFYYNSSTRRCEMFYYGGCGGNANNFNTLEECEKVCLGYGEAWKAP |

| Name | UniProtKB[a]/ Entry name | Organism | Family/ Scafold[b] | Coverage (%) | Mass (Da)[c] | Sequences of the polypeptides identified from the tryptic peptides obtained by MS /MS.[d] |
|---|---|---|---|---|---|---|
| DELTA-actitoxin-Aas1a | C5NSL2/ ACTP1_ANTAS | *Anthopleura asiatica* | Actinoporin family. Sea anemone subfamily. | 32 | 23,316 | MSRLIIAFIVVTMVCSAIALPKKKVEPLEKDEKRSLAVAGAVIEGGNLVMSVLDRILEAIGDVNRKIAIGVENQSGKSWTAMNTYFRSGTSDVVLPHSVPSGKALLYDGQKTRGPVATGVVGVFAYAMSDGNTLAVMFSIPYDYNLYSNWWNVKTYSGMKRADQSMYEDLYYHASPFKGDNGWHSRNLGYGLKCRGFMNSSGAAKLEIHVSRA |
| PI-AITX-Axm2b | P0DMX0/ VKT3_ANTAF | *Anthopleura aff. xanthogrammica* | Venom Kunitz-type family. Sea anemone type 2 potassium channel toxin subfamily. | 32 | 6,980 | HSSNDFCYLPAVRGRCRGYFPRYFFSSETGKCERFIYGGCGGNRNNFESAQECGSTCYPRE |
| Delta-actitoxin-Ael1c | P0C1F1/ NA13_ANTEL | *Anthopleura elegantissima* | Sea anemone sodium channel inhibitory toxin family. Type I subfamily. | 100 | 4,811 | GVPCLCDSDGPNVRGNTLSGTYWLAGCPSGWHNCKSSGPLIGACCKQ |

| Name | UniProtKB[a]/ Entry name | Organism | Family/ Scafold[b] | Coverage (%) | Mass (Da)[c] | Sequences of the polypeptides identified from the tryptic peptides obtained by MS /MS.[d] |
|---|---|---|---|---|---|---|
| Delta-actitoxin-Avd1e 3 | P0DL54/ NA126_ANEVI | *Anemonia viridis* | Sea anemone sodium channel inhibitory toxin family. Type I subfamily. | 58 | 8,677 | MMNRLLVFLMLGAAFMLVVSAIDQDANEDINKRGIPCLCDSDGPSVRGNTLS GIIWLAGCPSGWHNCKKHGPTIGWCCKQ |
| Sodium channel toxin protein | A0A0S1M143/A 0A0S1M143_AN ESU | *Anemonia sulcata* | Sea anemone sodium channel inhibitory toxin family. | 58 | 8,726 | MMNRLLVFLMLGAAFMLVVSAIDQDAYEDINKRGIPCLCDSDGPSVRGNTLS GIIWLAGCPSGWHNCKKHGPTIGWCCKQ |
| U-actitoxin-Avd8b | P0DMZ4/ TX8B_ANEVI | *Anemonia viridis* | Sea anemone 8 toxin family. | 12 | 8,863 | MKSLVIVFVVLLGVAMISANEEELLAILQDQRNDARGGCVNKYKRNICGTLVT PMNCIAPRTRMGKFARRFCKFMCGFC |
| Delta-actitoxin-Axm1f | P0C5G1/ NA14_ANTXA | *Anthopleura xanthogrammi ca* | Sea anemone sodium channel inhibitory toxin family. Type I subfamily. | 100 | 5,018 | GVPCLCDSDGPSVRGNSLSGIIWLFGCPSGWHNCRDHGPTIGWCCKK |

| Name | UniProtKB[a]/ Entry name | Organism | Family/ Scafold[b] | Coverage (%) | Mass (Da)[c] | Sequences of the polypeptides identified from the tryptic peptides obtained by MS /MS.[d] |
|---|---|---|---|---|---|---|
| Putative cell adhesion protein Sym32 | Q9NH96/ Q9NH96_ANTEL | *Anthopleura elegantissima* | Fasciclin | 58 | 32,956 | MKCILLASVVAVLIVSSYSLPLLELKPAVKLPKPSQNLGNFVEVANSLGLTDLISA AKAAGIAQFLVMGKSLTLFGPTNEAFDTIPEAYKPINSTFLKEVLLFHVIKSVVYA NAIKNELLVPSILEMPKKDIRFNVYGGGKIVTAQCSPIIKVNQNASNGVIHVVSR VMIPPFGTVTDVVAMEKQYFSTLLTAVLAAKLQGVLAGPGPFTVFAPTNEAFA KIPAEKLKEILKNIPLLTKILKYHVVSGTFCSAGLTNGATVPTLEGSDVTVHISGGS VTVNNAVVVFVDIPVTNGVVHVIDTVLIPKDVEV |
| Carbonic anhydrase | Q9XZG6/ Q9XZG6_ANTEL | *Anthopleura elegantissima* | Carb_anhydrase. | 69 | 28,612 | MAAPKWGYGPNNGPSKWAKDFPAAAGARQSPIDIKTHDAQHDSALKIKPLKI QYSQGNDFNVTNNGYSLVISRKTSEGTNLSGGPLEHNYRFEQFHFHWGKTSG SGSEHLLDGKAFPAELHLVHWNTDLFSSFGEAASSKNGLAVLGAFVQIGGESA GLKTITDLIPQVQNIGDKQDLKVPFNLSSLLPSNTNDYWTYSGSLTTPPCYESVS WFVFKEPIHATENQMQQFRSLKANDGGCIVDNYRPVMDGSGRNVRASFE |
| Superoxide dismutase [Cu-Zn] | Q2F6F3/ Q2F6F3_ANTEL | *Anthopleura elegantissima* | Cu-Zn superoxide dismutase family. | 91 | 15,725 | MVVKAVCCLVGDVKGTINFTQEGDGKPCHITGEVTGLTEGKHGFHIHQYGDN TNGCTSAGSHFNPFGKNHGGPDDTDRHVGDMGNIVAGKDGVGKVDMKEN QVTLLGEHSVVGRSVVVHVGEDDLGKGGHDDSLTTGHAGGRLACGVIGICPV |
| Methylmalonate semialdehyde dehydrogenase-like protein | Q2F6F8/ Q2F6F8_ANTEL | *Anthopleura elegantissima* | Aldehyde dehydrogenase family. | 28 | 59,416 | HETFTCGSHLIKNSFIFFLTMYRIIPLSRKVSPLSVACRRYMTNGTPATTKLLING QFIESKTSKWIDLHNPATNEVITRVPESTPEEMQAAVDAASAAFPSWSETSILA RQQIMFKFQQLIKENMKELAHNITLEQGKTLPDAEGDVLRGLQVVEHTCSITS LQMGETMQSVTKDMDTYSYRTPLGVCAGITPFNFPAMIPLWMFPVAIVCGN TYVIQALRKRSWCMYDAVKDGTDSGLPDGVVNVIHGARDAVSFICDDPAIKAI SFVGSDQAGRFIYERGSKSGKRVQSNMGAKNHGVIMPDASKESTINQLVGAA FGAAGQRCMALSTAIFVGETKEWIPEIVEKARKLQVNAGDQPGADLGPVISP QSKNRICDLVQSGVEEGAKMELDGRDVVVKGYENGNFVGPTILSGVKPEMKC YTEEIFGPVLVAMDCDTMEDAIEIINRNPYGNGTAIFTNSGAVARKFQQKVDV GQIGVNVPIPVPLPMFSFTGSRGSFLGDAHFYGKQGVNFYTGTKTITSLWRDE DAKPQKLQAAFPTIK |

| Name | UniProtKB[a]/ Entry name | Organism | Family/ Scafold[b] | Coverage (%) | Mass (Da)[c] | Sequences of the polypeptides identified from the tryptic peptides obtained by MS /MS.[d] |
|---|---|---|---|---|---|---|
| Myosin regulatory light chain 2-like protein | Q2F6G8/ Q2F6G8_ANTEL | *Anthopleura elegantissima* | EF-hand | 25 | 19,602 | MSSKAKDKKKGGTKKKAQRATSNVFAMFDQQQIQEFKEAFNMVDQNRDGF ISKDDLAATFDSLGKLVNDEFLEEMLGEATGPVNFTMFLTLFGEKISGTDPEDVI RHAFSSFDPENQGFIDESKLKRLIQGLGDRFTEEEWDMMMDDCPVNKKGDIL YNDFVHLIKYGPKED |
| CnidEF (Fragment) | Q2ESH7/ Q2ESH7_9CNID | *Anthopleura artemisia* | EF-hand | 43 | 13,851 | VRVRRCYLACSPTKCRIICVVSYHVVCPGKRDEIEDAQGSRKKRPEKAIIEKFSNK FSTYDKDGDNSISFDEFRTTLGDVHDRKMLRNLFNTTDKNGDNAITCDEFLKA KFDFSEKPVCQ |
| Oncoprotein-induced transcript 3-like protein | Q2F6H3/ Q2F6H3_ANTEL | *Anthopleura elegantissima* | Zona_pellucida | 29 | 17,539 | TEGYDSKPQYVFINNSCGQDDTLKYDFKESGVQRFSLAAFRFLP DFKEVYLHCRVVACPGGQSDSRCAVGCKNSGGSGRRRRAATL VRDIEQDLALGPIKLTDRQESSAQAASSGSMITMVTIVAGVLGF LVLCLVIAIVIIYKRKQSHTNGAKLLVQQEA |
| Death-associated protein 1-like protein. (Fragment) | Q2F6H1/ Q2F6H1_ANTEL | *Anthopleura elegantissima* | DAP | 66 | 7,311 | RENEEFAEPKERDSTKLVVSGAVAKGNADFPPDAVKVAHEKPI PQHDKRPPGAGGKGANISIQQPRKN |
| Beta-actin (Fragment) | Q2LGN4/ Q2LGN4_ANTEL | *Anthopleura elegantissima* | Actin | 38 | 14,948 | SLEKSYELPDGQVITIGNERFRCPEAMFQPSFLGMESAGIHETCY NSIMKCDVDIRKDLYANTVLSGGSTMYPGIADRMQKEIANLAP PTMKIKIIAPPERKYSVWIGGSILASLSTFQQMWISKQEYDESGP S |
| Peridinin-chlorophyll-protein | Q944P5/ Q944P5_SYMMU | *Symbiodinium muscatinei* | PCP | 49 | 20,867 | MAMKVRAAGLLLLLSFCLSCVVFVPGPRHVGPVAAGALGMM AAAPAYADKIDDAAKVLSEKSYPFLKEIDWTSDVYAKLPTQKP VAVMEAINKMLVMGAAMDSEALKKGVLAHAKAIDGMDSKL VATLDDYTAINSAIGHMIASVPASKTMDVYNAFAKFSLGSDVG PYMMSKVNAADAKAAYEALLAFKDVVKASQR |
| Fructose-bisphosphate aldolase. (Fragment) | C7SP12/ C7SP12_ANESU | *Anemonia sulcata* | Glycolytic | 77 | 21,656 | DESTGTMGKRLANISVENSEENRRQYRQLLFTSGKEMSNAISR ALLFEETLYQKADDGTPFVKIIRDQGIIPGIKVDKGVVPLAGTV GEGTTQGLDGLSQRCEQYKKDGCDFAKWRCVLKITDHTPSEL AIKENANVLARYATICQQNGIVPIGEREVLCDGDHSLERAQKVT EAVLSAQYKALVDHHVYLEGSLL |

| Name | UniProtKB[a]/ Entry name | Organism | Family/ Scafold[b] | Coverage (%) | Mass (Da)[c] | Sequences of the polypeptides identified from the tryptic peptides obtained by MS /MS.[d] |
|---|---|---|---|---|---|---|
| Glyceraldehyde-3-phosphate dehydrogenase. (Fragment) | A8DX89/ A8DX89_URTEQ | *Urticina eques* | Glyceraldehyde-3-phosphate dehydrogenase family. | 28 | 31,988 | GRLVFRASLERDDVQLVAVNDPFLDAEYMAYLFKYDSTHGRFKGDVKVADGKLIINGKTVEVYAERDPTKIPWGKHGADTVVESTGVFTTIEKAGAHLEGGAKKVIISAPSADAPMFVMGVNEGKYENTMNVVSNASCTTNCLAPLAKVINDNFGIVEGLMTTIHAYTATQKTVDGPSGKKWRDGRGANQNVIPASTGAAKAVGKVIPELNGKLTGMAFRVPVPDVSVVDLTVRLKNPASYEKIKSVVKAASESKEMGRYLGYTEDDVVSSDFIGDTTSSIFDAKAGLALNDNFVKLVT |
| Elongation factor 1-alpha. (Fragment) | Q16995/ Q16995_ANEER | *Anemonia erythraea* | GTP_EFTU | 41 | 44,969 | GHLIYKCGGIDKRAIEKFEKEAAEMGKGSFKYAWVLDKLKAERERGITIDIALWKFETNKYYVTVIDAPGHRDFIKNMITGTSQADCTVLIVASGTGEFEAGISKNGQTREHALLAFTLGVKQMIVCCNKMDNTESTLREARFQEIEKEVGSYLKKIGYNPKKIHFVPISGWHGDNMLEKSDKMPWWNGFELFNKSQGSKTGTTLFDGLDDINVPSRPTDKALRLPLQDVYKIGGIGTVPVGRVETGILKPGMLVTFAPVNLTTEVKSVEMHHTALTEALPGDNVGFNVKNLSVKDIKRGFVAGDSKNDPPKNIGFFDAQVIVLNHPGEIHNGYSPVLDCHTAHIACKFYEIKNKGDKRSGKVTEEFPKAIKSGDAALVTLQPSKDMCVEKYSSYAPLGRFAVRDM |
| Blue chromoprotein aeCP597 | Q2VFP3/ Q2VFP3_ACTEQ | *Actinia equina* | GFP family | 26 | 25,913 | MASLVKKDMCIKMTMEGTVNGHHFKCVGEGEGKPFEGTQVEKIRITEGGPLPFAYDILAPCCMYGSKTFIKHVSGIPDYFKESFPEGFTWERTQIFEDGGYLTIHQDTSLQGNNFIFKVNVIGANFPANGPVMQKKTAGWEPCVEMLYPRDGVLCGQSLMALKCTDGNHLTSHLRTTYRSRKPSNAVNMPEFHFGDHRIEILKAEQGKFYEQYESAVARYCEAAPSKLGHH |
| GFP-like non-fluorescent chromoprotein | Q95W86/ NFCP_CONGI | *Condylactis gigantea* | GFP family | 28 | 25,416 | MAGLLKESMRIKIYMEGTVNGYHFKCEGEGDGNPFEGTQNMRIRVTEGAPLPFAFDILSPCCAYGSKTFIKHTSGIPDYFKQSFPEGFTWERTTIYEDGGVLTAHQDTSLEGNCLIYKVKVLGTNFPADGPVMKKISGGWEPCTEIVYQDNGVLRGRNVMALKVSGRPPLICHLHSTYRSKKACALTMPGFHFADLRIQMPKKKKDEYFELYEASVARYSDVPEKAT |

| Name | UniProtKB[a]/ Entry name | Organism | Family/ Scafold[b] | Coverage (%) | Mass (Da)[c] | Sequences of the polypeptides identified from the tryptic peptides obtained by MS /MS.[d] |
|---|---|---|---|---|---|---|
| Mitochondrial 60 kDa heat shock protein | Q6RFF9/ Q6RFF9_ANEVI | *Anemonia viridis* | Chaperonin (HSP60) family. | 63 | 62,807 | MYRLPSLIRPGRLVLSSRSLVPRLGASFSTSPQQNAKELKFGADARSSMLQGVEVLADAVAVTLGPKGRNVIIEQSFGGPKITKDGVTVAKAIELKDKFQNIGARLVQDVANNTNEEAGDGTTTATVLARSIATEGFSKVSKGANPQEVRRGVMLAVENIVDSLKQMSKPVTTPEEIAQVATISANGDKRIGELISSAMKRVGRSGVITVKDGKTLNDEMEVIEGMKFDRGHISPYFINTAKGQKVEYQDCLVLLCQKKISSIQQIVPALELANSHRKPLVIVAEDVDGEALTTLVLNRLKVGLQIAAVKAPGFGDNRKNMLQDMAIATGGMVFGDEALETKLEDIQIQDFGEVGEVSITKDDTLFLRGKGSQEDVEKRCDHIKEELDSTNSEYEKEKLNERLAKLSDGVAILKIGGSSEVEVNEKKDRVTDALNATRAAVEEGIVPGGGVALLRRTNNLNDLKLENAEQEIGVELVIKALRKPLHTIAENAGVEAALVVEKVLQQNGNSGYDAQNNKYVDMIQEGIIDPTKVVRTAITDAAGVASLLTTAETVIVEAPKDEKDPMAGMGGMGGMGGMGGMGGMGGMM |
| Catalase | A0EJ86/ A0EJ86_ANEVI | *Anemonia viridis* | Catalase family. | 22 | 57,366 | MASRTKASEQMSQFAQAQKGQDVLTTSGGNPVDTNTSTMTVGPRGPVLMQDTQYMDVMSHFDRERIPERVVHAKGGGAFGYFEVTHDISKYCKAKIFEKIGKTTPCLLRFSTVGGESGSADTVRDPRGFALKFYTEEGNWDLVGNNTPIFFIRDPILFPSFIHTQKRNPVTHLKDPDMFWDFITLRPETTHQVCFLFSDRGIPDGHCHMNGYGSHTFKMVNDAGEAVYCKFHLKTDQGIKNVPVEVASRLSGEDPDYSNRDLYNRIAGGKYPSWTMYIQVMTFDEAEKFSFNPFDLTKIWPHEEFPLIPVGKMVLNRNPKNYFAEIEQSAFNPANMVPGIEASPDKMLQGRLFSYHDTHLHRLGTNYTQLPVNCPFSTRVRNYQRDGPQTFDNQEGAPNYFPNSFSGPVDNAKFTPSAFKLSGDVARYNSADEDNFTQVTTFWTKVLNDEERGRLVRNIAGHVKDAKEFIQNRCVANFMQVHPDFGNGIAKELAKYKQGNVGAGAHAAL |

| Name | UniProtKB[a]/ Entry name | Organism | Family/ Scafold[b] | Coverage (%) | Mass (Da)[c] | Sequences of the polypeptides identified from the tryptic peptides obtained by MS /MS.[d] |
|---|---|---|---|---|---|---|
| Glyceraldehyde 3-phosphate dehydrogenase.(Fragment) | C7SP37/ C7SP37_ANESU | *Anemonia sulcata* | Glyceraldehyde-3-phosphate dehydrogenase family. | 40 | 20,557 | KTVEVYANKDPSTIPWGSHGADTVIESTGVFTTIEKAGAHLKGGAKKVIISAPSADAPMFVMGVNEEKYETSMNVVSNASCTTNCLAPIAKVINDNFGIVEGLMTTIHAYTATQKTVDGPSGKKWRDGRGAHQNVIPASTGAAKAVGKVIPELNGKLTGMAFRVPVPDVSVVDLTVRLKNGASYDQIKSVVKKASE |
| Caspase 3-like protein | Q09Y99/ Q09Y99_ANEVI | *Anemonia viridis* | Peptidase C14A family. | 15 | 45,753 | MEQIHRDALRSNRQALIQDLEASKIASNLYGTGILDENDKDKVNAGDTNADRAEKLLDILPRKGPKAFNAFCDALKDISPHLEQLINPATTQGKEAETDDRVTRTSVHPRSVASVTTSSGAIGGKVPITASEDGPSESSGDNPDGGLNIFGSGSSRPRPSEPIDIRSMYKMDKSPRGMAIIINNKTFLPSSGMHRYPRNGTDVDRDALEKTFNRLMFNTLVYNNQSVYETQKIFKGLATKDFSNENALVVCILTHGEEGILYATDGTILIKDMMGWFKGSNLVGKPKVFIFQACQGHEYMDGKDATDAPPPDKRVQIPVEADFLYAYSTVPGYYSWRNSVNGSWFIQSIVEVFNKYAKTTDLLTMMTRVNALVATYQSRTNDPYSDRKKQIPSIVSMLRKDLYFFPNNLVDS |
| Niemann-Pick C 2 Like | W0Z6N1/ W0Z6N1_ANEVI | *Anemonia viridis* | ML domain - MD-2-related lipid recognition domain. | 20 | 16,168 | MKFLVLLLCLQIIWSLSEARKLSFKDCGSKVGKLVSFDLSPCSQDPCIIKRGSNATGTVTFIPSEEVTSSKVYMYAIIGFIPVPLPLPNTDGCKGYGLTCPLKSGKPDELVFSHSIDSTFPAGTVTLKGELKDQEENNIFCGKISLTLQ |
| Blue chromoprotein 2 | A0A1D7XF02/A0A1D7XF02_ACTTE | *Actinia tenebrosa* | GFP | 50 | 25,966 | MSSLVKKDMCIKMTMEGTVNGHHFKCVGEGEGKPFEGTQVEKIRITEGGPLPFAYDILAPCCMYGSKTFIKHVSGIPDYFKDSLPEGFTWERTQIYEDGGYLTIHQDTSIQGDSFIFKVKVIGANFPANGPVMQKKTAGWEPCVEMLYPRDGVLCGQSLMALKCTDGNHLTSHLRTTYRSRKPANAVNMPKFHFGDHRIEILKEAEPGKFYEQYESAVARYCEAAPSKLGHH |

| Name | UniProtKB[a]/ Entry name | Organism | Family/ Scafold[b] | Coverage (%) | Mass (Da)[c] | Sequences of the polypeptides identified from the tryptic peptides obtained by MS /MS.[d] |
|---|---|---|---|---|---|---|
| Myosin heavy chain type II. (Fragment) | Q8MV94/ Q8MV94_AULVE | *Aulactinia verrucosa* | Myosin family. | 10 | 23,001 | LQQFFXHHMFVLEQEEYKREGIHWEFIDFGLDLLPTIDLIFSKGTGIFALLEEECIVPKATDQTLLAKLNSTHDGKSVKFGKPKISGKKTINYHFEIHHYAGSVGYNIDNWLDKNKDPINEAVASIFAKSSDPFIAGLWKDYATEGHRGKGGSFMTVSAKHKEQLNKLMDTLYSTAPHFVRCIIPNELKKAGVIDTNLVLHQL |
| Histone H3. (Fragment) | C7SNW9/ C7SNW9_ANESU | *Anemonia sulcata* | Histone H3 family. | 7 | 10,246 | GGKAPRKQLATKAARKSAPSTGGVKKPHRYRPGTVALREIRRYQKSTELLIRKLPFQRLVREIAQDFKTDLRFQSAAIGALQEAAEAYLVGL |

[a]Uniprot access number.

[b,c,d]Data obtained from the UniprotKB database.

[d]The region of the amino acid sequence covered by the tryptic peptides obtained from F1 is highlighted on a gray background.
